# The First District Dental Society Meeting and Some of Its Results

**Published:** 1887-02

**Authors:** 


### Editorial.

### THE FIRST DISTRICT DENTAL SOCIETY MEETING AND SOME OF ITS RESULTS.

The eighteenth anniversary meeting of the First District Dental Society of the State of New York was a memorable occasion for more reasons than one. First, it was, if all accounts be correct, the largest purely dental society meeting ever held in America, the number in attendance being about six hundred. The exercises, too, were of an unusual nature, the clinics being a principal feature and the meeting of a decidedly practical character. But that which, perhaps, is of greatest moment to dentists as a body, was not laid down in the programme, and occurred quite unexpectedly. We refer to the complete breaking down of the opposition to the dental section of the Medical Congress which is to meet at Washington, this year.

There has been a deep undercurrent of dissatisfaction with the organization of the Medical Congress, especially among the dentists who are connected with medical societies, ever since the annual meeting of the American Medical Association at New Orleans, when that society assumed the management of the Congress, through its committees. There were those who did not believe that any single society should arrogate this authority, inasmuch as it had always been the policy of the Congress to keep itself wholly aloof from society matters, and to recognize no professional organization whatever. It is not a representative body, but is a convention of the medical men of the world. The section of dental and oral surgery, as first instituted, was, under the New Orleans reorganization, dropped, or what was the same thing, was consolidated with another purely medical section, and entirely lost its dental character. This intensified the feeling of the dentists who did not approve the revolutionary proceedings at New Orleans, and when it was again established it was believed, whether correctly or incorrectly, that it was not done spontaneously, but because the prospects for the Congress were gloomy and the assistance of the dentists was sorely needed. The organization of the dental section proceeded but slowly, when the Buffalo conference was called and the situation discussed. The decision at that meeting was that it was inexpedient, under the then existing circumstances, to proceed with the formation of a dental section, and the most of the officers who had then been appointed sent in their resignations.

But the president of the section, Prof. Taft, did not lose confidence in the undertaking, and held on, although little was done for some time in urging the movement forward. There was no active opposition, but because they were unfavorably prepossessed there existed a most decided coolness and apathy on the part of many of the leading men in dentistry. The feeling, however, among those who had not identified themselves with the section was, that it would be unwise and unfair to throw any obstacles in the way of those who desired a meeting. It was the undoubted privilege of any respectable number of dentists to hold any meeting which they desired, and those in favor of the section were something more than a mere respectable portion of dentistry. They had certain rights, and even had they been in the minority, those rights must and should be respected.

There has always existed a difference of opinion among dentists as to the true status of dentistry in its relation to medicine, some contending that a medical education was comparatively unnecessary and undesirable, others believing that it should form the foundation for a special training in a specialized medical practice. But the question had never become a living issue, for the time had not arrived when it was necessary to make a distinct departure and to choose a path which should plainly lead either directly away from or towards a more intimate connection with the great mother profession.

This was the condition of affairs when, in October last, at a meeting of the New England Dental Society, Dr. N. W. Kingsley, of New York, read a paper entitled "Dentistry Not a Specialty of Medicine," in which it was urged that there was really no more intimate connection between the two than between any other of two distinct avocations. His views were urged with all the ability, the confidence and the aggressiveness which distinguish the papers of the author. A part of the address was devoted to the consideration of the advisability of sustaining the dental section of the coming Congress, and the author took the ground that as an independent profession we had no business there, and that no considerations urged us as dentists to take any part in the Congress. He urged that our interests lay in the formation of an International Dental Congress, to be held in the near future. This was the first outspoken and open opposition which had been given voice, for the organization of an International Dental Congress at this time could be interpreted in no other way than as a direct blow at the dental section of the Medical Congress.

The paper was read before a number of different societies, and resolutions were at each meeting offered, looking toward the organization of a dental congress. It was published in full in the last number of this journal, and in an editorial we presented the reasons why this was done. We felt and said that the question at issue should now be met and settled. There should be a fair consideration, and an intelligent expression of the views of the best men in dentistry should be obtained, and we intimated that the pages of this journal were open for a calm discussion of the matter. A very complete answer to Dr. Kingsley's paper was read before the Brooklyn Dental Association, by Dr. C. A. Marvin, and that paper will be found in this number. Another paper from a more purely medical stand point, by Dr. Herbert M. King, will also be found on another page.

But it was felt that at the anniversary meeting of the First District Society the question would probably reach some kind of a solution. Neither of the authors of the papers published, we think, looked for such a speedy and complete settlement of the whole matter as was attained there, for although there was no debate over the matter, it was most definitely and positively decided. The current of opinion was so unmistakably and peremptorily opposed to the views of Dr. Kingsley, that there was nothing to do but to make a virtue of necessity and yield the point as gracefully as possible, and this he did, withdrawing and disclaiming any opposition to the Congress, and pledging to it his hearty and earnest support in the future.

Perhaps there never was a gun fired, the recoil of which was so instant and tremendous as the one of which the final report was heard in our last number. It produced results for which the energetic President, with all the officers and friends of the dental section of the Congress, had labored for nearly two years in vain. As long as the section was not attacked openly it needed no defenders, and there was an unmistakable indifference in many quarters as to what it accomplished or failed to do. But when danger menaced it, when it was wounded in the house of its supposed friends, the chivalry of dentists was aroused, and those who had retired to their tents came out in full armor, ready to do battle in its cause. The reaction was as powerful as unexpected, and at the final winding up of the First District meeting at Mazettis', a general love-feast was indulged in, and all agreed to bury past differences, sink personal feelings and join hands to make of the dental section of the Ninth International Medical Congress such a success as dentistry has never known. There were mutual concessions and apologies. It was agreed that, perhaps, mistakes had been made on all sides, but that now was the time to forget the things which were behind, looking only to that which is before us.

Dr. Kingsley took the wise and judicious course. He found himself on the wrong side of the fence, and lost no time in getting on the right side, as every honest and conscientious dentist who is earnestly laboring for the highest good of his profession should do. There were others who have thus far stood aloof, declining any active participation in the section, who have now promised their hearty co-operation, and who will give to the section their best and most earnest efforts. The result must be particularly gratifying to the persistent president of the section, who has labored in the face of the utmost discouragement, but who now sees the dentists practically united in support of the cause which is so near his heart, and he is to be heartily congratulated on the result.

And this is why we said that the matter which, in connection with the First District Society meeting, was of greatest import to dentists, was not laid down in the very elaborate and extensive programme that was published.

We have thought it best to thus briefly review the history of the dental section, that the changed situation of affairs might be thoroughly comprehended. For one, we do not pretend to like the medical organization of the Congress any better than heretofore, but that is a matter with which we as dentists need have nothing to do. It only remains for us to so manage our section that we shall win the applause of all, whether in or out of the Congress.

